# Preoperative diagnosis of hiatal hernia: barium swallow X-ray, high-resolution manometry, or endoscopy?

**DOI:** 10.1007/s10353-017-0492-y

**Published:** 2017-09-19

**Authors:** Michael Weitzendorfer, Gernot Köhler, Stavros A. Antoniou, Leo Pallwein-Prettner, Lisa Manzenreiter, Philipp Schredl, Klaus Emmanuel, Oliver Owen Koch

**Affiliations:** 1 Department of Surgery, Paracelsus Medical University, 5020 Salzburg, Austria; 2 Department of General and Visceral Surgery, Ordensklinikum Linz Sisters of Charity Hospital, Linz, Austria; 3 Department of General Surgery, 401 Military Hospital, Athens, Greece; 4 Department of Diagnostic and Interventional Radiology, Ordensklinikum Linz Sisters of Charity Hospital, Linz, Austria

**Keywords:** Hiatus hernia, Endoscopy, High-resolution manometry, Barium swallow, Gastroesophageal reflux disease

## Abstract

**Background:**

The assessment of hiatal hernias (HH) is typically done with barium swallow X‑ray, upper endoscopy, and by high-resolution esophageal manometry (HRM). The aim of this study was to assess the clinical utility of these methods in terms of HH detection and their correlation to gastroesophageal reflux disease (GERD).

**Methods:**

A retrospective comparative analysis of patients with symptoms of GERD was carried out. The performance of endoscopy and HRM in diagnosing HH was assessed, taking barium swallow X‑ray as a reference. Furthermore, statistically comparative analysis between detected hernias and the presence of reflux disease in ambulatory impedance-pH monitoring (MII) was performed.

**Results:**

Overall, 112 patients were analyzed. Barium swallow X‑ray showed no correlation either to HR manometrically or to endoscopically assessed HH. Significant accordance in the detection rate of HH was proved between HRM and gastroesophagoscopy (*p* < 0.001). Only endoscopically assessed HH showed a significant correlation with GERD (*p* = 0.047). No correlation between detected hernias and GERD could be found either with HRM or with barium swallow X‑ray.

**Conclusions:**

Barium swallow X‑ray provided the highest rate of HH detection (76.8%). For the reliable exclusion of HH prior to treatment, all three mentioned investigations appear to be necessary in order of low conformity.

## Introduction

Hiatus hernia (HH) is recognized as an important factor in the pathophysiology of gastroesophageal reflux disease (GERD). The presence of HH is associated with GERD symptoms [1, 2]. HH develop in 10%–50% of the general population and can be classified into four types: Type I is the sliding hernia which is most common and accounts for 85% of cases. It is defined as cephalad migration of the esophagogastric junction through the esophageal hiatus [3, 4]. The diagnosis of sliding HH is commonly made with upper gastrointestinal endoscopy, barium swallow X‑ray, and esophageal manometry [5–8]. Sliding HH can be characterized at endoscopy, when the diaphragmatic indentation is seen 2 cm or more distal to the squamocolumnar junction (the so-called Z‑line) and the top of the stomach mucosal folds [5, 9, 10]. Another approach is to assess the appearance of the esophagogastric junction from a retroflexed position and to incorporate an assessment of hiatal integrity along with the assessment of axial displacement. The progression from normal anatomy to type I hernia was well illustrated in an analysis of “flap valve” integrity as a predictor of reflux symptoms. The role of an altered geometry of the gastroesophageal flap valve (GEFV) in the pathophysiology of GERD was recognized years ago. Since its first description in 1996 by Hill et al. [9], several studies have shown that the retroflex grading of the GEFV is a simple and reproducible tool that provides additional information to correctly diagnose the patient’s status with GERD [11]. Radiologic diagnosis depends on characterization of the landmarks of the gastroesophageal junction extending above the diaphragmatic hiatus. Classic criteria include herniation of at least 2 cm of gastric cardia above the hiatus [12]. Contrast studies seem to be more sensitive than endoscopy in detecting sliding HH [1, 5, 13, 14]. With the relatively new high-resolution manometry (HRM) technology, the sliding component of HH can be detected and its size calculated. HRM may offer advantages in diagnosing HH over endoscopy and barium swallow X‑ray [15, 16]. The aim of this study was to assess the clinical utility of barium swallow X‑ray, endoscopic abnormal flap valve and HH assessment, and HRM in the diagnosis of sliding HH in patients with GERD symptoms. Since the presence of HH is associated with GERD, we were also interested to discover whether there are differences in objective GERD detected by ambulatory multichannel intraluminal impedance-pH monitoring (MII) and the HH diagnosed using the other investigations.

## Material and methods

A retrospective analysis of 112 consecutive patients with subjective symptoms of GERD who invariably underwent gastroscopy, HRM, barium swallow X‑ray, and MII after pausing proton pump inhibitors (PPI) for 2 weeks between August 2012 and October 2013 at our institution (Sisters of Charity Hospital Linz, Austria, Departments of General and Visceral Surgery and Diagnostic and Interventional Radiology) was performed. All patients reported persistent or recurrent symptoms of GERD despite therapeutic treatment with PPI for a minimum duration of 6 months. Data were collected from the hospital electronic database and medical records. Patients with paraesophageal hernias (HH type III–IV), achalasia, and other primary esophageal motor disorders, as well as those with previous fundoplication, were excluded.

Study approval was obtained from the institution’s ethical committee.

### High resolution esophageal manometry

We used the ManoScan esophageal manometry system from Given imaging™. Analysis of gastroesophageal junction morphology was performed using HRM topographic pressure plots as per the criteria suggested by Kahrilas et al. [10]. Three different subtypes can be distinguished according to these criteria. The Type-III morphology is consistent with >2 cm separation between the crural diaphragm and lower esophageal sphincter (LES). Only patients with Type-III morphology were considered to have an overt sliding HH. The manometry studies were done in a 30° supine position. Analysis was performed by one of the authors (O.O.K.), who was blinded to the radiographic and endoscopic findings at the time of analysis.

### Radiographic barium swallow examination

Barium swallow X‑ray was performed according to a protocol of five swallows of barium always using the same amount of liquid; anteroposterior and oblique views were obtained in upright and supine positions. A single radiologist (author L.P.P.), blinded to the endoscopic and manometric findings, retrospectively assessed the size of the HH in millimeters. By reviewing still images, measurements were done using a standardized protocol, according to which a distance of more than 2 cm between the gastroesophageal junction and the diaphragmatic hiatus was defined as a sliding HH [10, 12].

### Esophagogastroduodenoscopy (upper endoscopy)

**Fig. 1:**
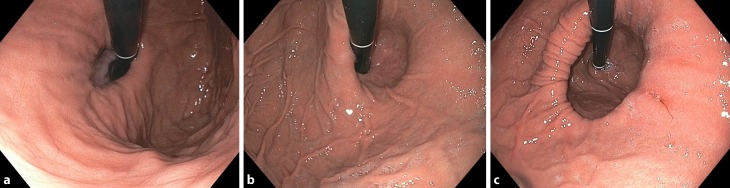
Examples of different sized hiatal hernias in esophagogastroduodenoscopy (**a** small; **b** medium; **c** large)

With effect from August 2012, all endoscopists in the department were encouraged not only to describe a HH (Fig. 1), but also to assess the gastroesophageal junction in a retroflexed position and grade it from I to IV according to the grading system described by Hill et al. [9]. Patients whose endoscopic findings were classified as Hill grade I and II were considered to have a normal and grade III and IV to have an abnormal GEFV. Only if endoscopists described a sliding hernia (separation of the squamocolumnar junction = Z‑line from diaphragmatic impression >2 cm) and an abnormal valve were the patients concerned included.

### Ambulatory multichannel intraluminal impedance-pH monitoring

Patients were encouraged to maintain their normal activities and mealtimes and to remain upright during the day except one short nap. All patients underwent 24-h ambulatory pH monitoring with a Given Imaging Digitrapper® pH Z Monitoring System (Yoqneam, Israel). The probe was placed 5 cm above the proximal border of the lower esophageal sphincter established by esophageal manometry. We used the symptom index (SI), i.e., the number of symptoms associated with reflux events based on a 5-min time window divided by the total number of symptoms. SI was declared positive if it was higher than 50% [17]. GERD was diagnosed if the total number of reflux events in 24 h exceeded 73, when the reflux-related composite pH score according to DeMeester exceeded 14.7, or if SI was positive for symptoms reported at least three times [18].

### Statistics

Statistical analysis of data was performed using SPSS statistical analysis software (SPSS Inc., Chicago, IL, USA). All data were tested for normal distribution by the Shapiro-Wilk test. Comparison between data sets was done using non-parametric tests and t‑test for paired samples. Analysis of data was also performed using chi-square test, Mc Nemar test, Fisher’s exact test, and correlation analysis using the Spearman’s test. Exact binomial statistics for conditional and marginal distributions and the kappa test for accordance of measurements were used. All data were presented as means with ranges or standard deviation (SD). A *p*-value of less than 0.05 was regarded as significant. Descriptive statistics were used in some cases.

## Results

In all, 112 patients (52 female, 60 male) with a mean age of 53.60 (SD ± 16.03) years and a mean body mass index (BMI) of 26.08 (SD ± 4.31) kg/m^2^ were retrospectively analyzed. With HRM, HH was diagnosed in 35 patients (31.25%) with a mean size of 30.51 mm (range, 20–61 mm), with radiology 86 patients (76.78%) showed a hernia with a mean size of 32.98 mm (range, 21–75 mm). In 54 patients (48.2%), an abnormal GEFV/HH was diagnosed with upper endoscopy.

**Fig. 2:**
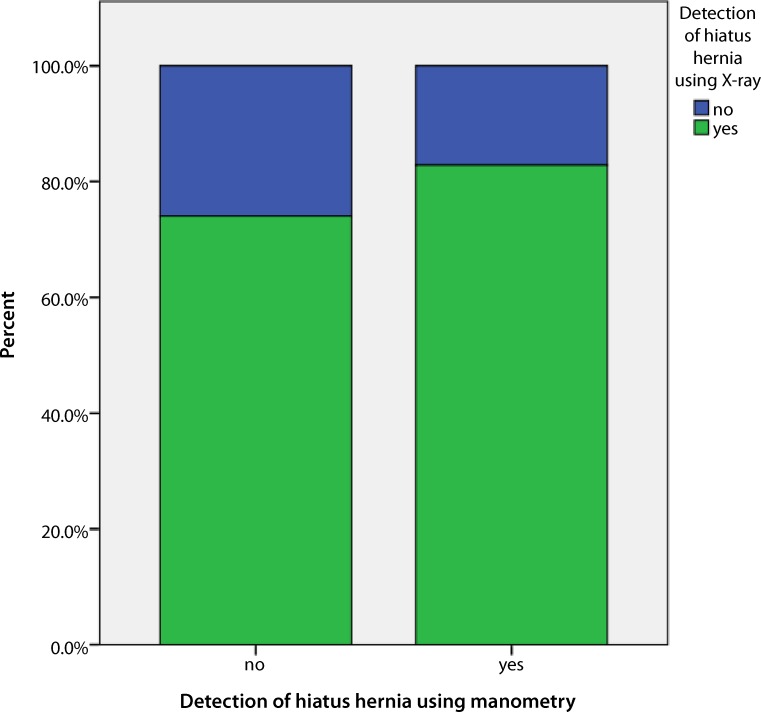
Cross-tabulation diagram (bar chart) of manometric (HRM) and radiographic hiatus hernia detection

There was no significant equivalence and accordance between HRM and barium swallow X‑ray regarding the detection rate of HH. Of 77 patients that had no hernia on HRM, 57 (74%) showed a hernia in the barium swallow X‑ray examination (Kappa test for accordance of measurements: *p* = 0.30). There was a high significance for discordance of paired samples by using the McNemar test (*p* < 0.001). The Spearman’s rank correlation test also showed no correlation between the two compared methods regarding HH detection (*p* = 0.309). Fig. 2 outlines the discordance in a cross-tabulation bar chart: the green part of the left-sided bar shows no HH detection in manometry, but detection in radiography (74%) with an accordance of only the remaining 26% with no HH detection in manometric and radiographic findings.

**Fig. 3:**
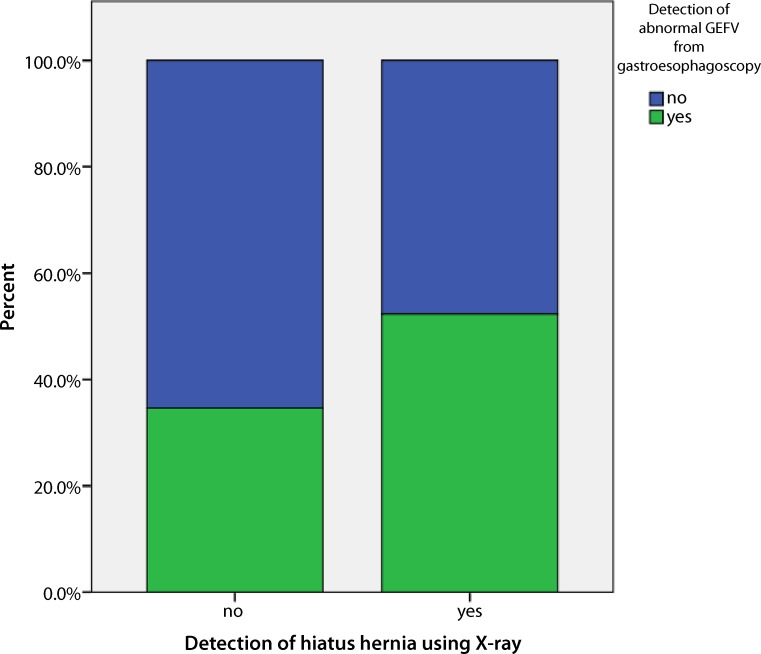
Cross-tabulation diagram (bar chart) of endoscopically abnormal gastroesophageal flap valve/hiatal hernia (HH) and radiographic HH findings

There was also no correlation according to the hernia detection rate in barium swallow X‑ray and endoscopic measurements (Kappa: *p* = 0.11, McNemar for discordance of paired samples: *p* < 0.001, Spearman’s rank correlation: *p* = 0.115). The cross-tabulation bar chart is shown in Fig. 3.

A comparison of the HH detection rate between HRM and endoscopy showed a significant accordance of measurements (Kappa: *p* < 0.001, Spearman *p* < 0.001).

Objective GERD was diagnosed with MII in 77 patients (68.8%). This was the number of patients with pathological findings as constituted in the methods section. In all, 28 of 35 patients (80%) with a manometrically detected hernia had diagnosed GERD, 63/86 patients (73.25%) with radiologically detected hernia, and 46/54 patients (85.18%) with endoscopically detected abnormal GEFV/HH showed objective GERD in pathological MII evaluation.

**Fig. 4:**
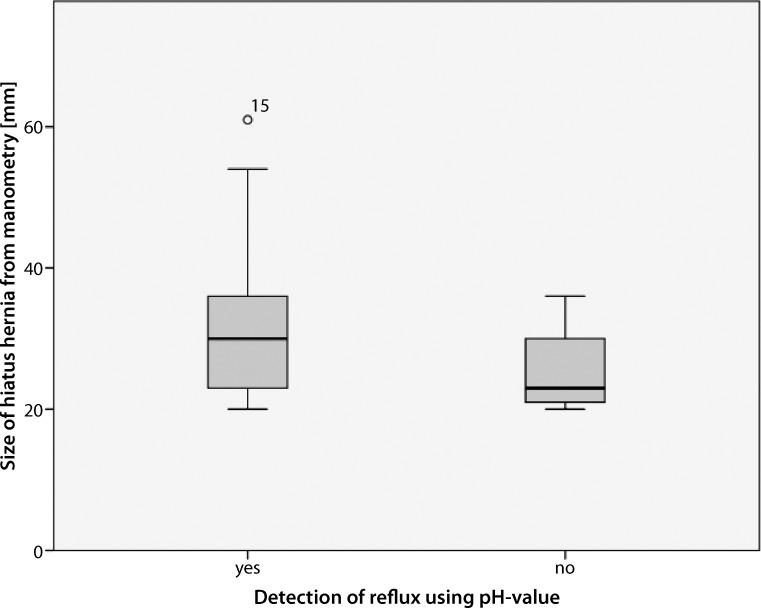
Box-plot diagram of manometric hiatus defect size and pathological ambulatory multichannel intraluminal impedance-pH monitoring

There was no general correlation between the HH detection rate in HRM and objective GERD (Spearman: *p* = 0.683). Nevertheless, the non-parametric Mann-Whitney-U test showed a significant correlation between manometrically measured HH size (not to be confused with the HH detection rate) and objective GERD in MII evaluation (*p* = 0.031). Fig. 4 shows the mean manometrically measured HH sizes in patients with and without pathological MII findings (32.40 mm vs 25.80 mm).

**Fig. 5:**
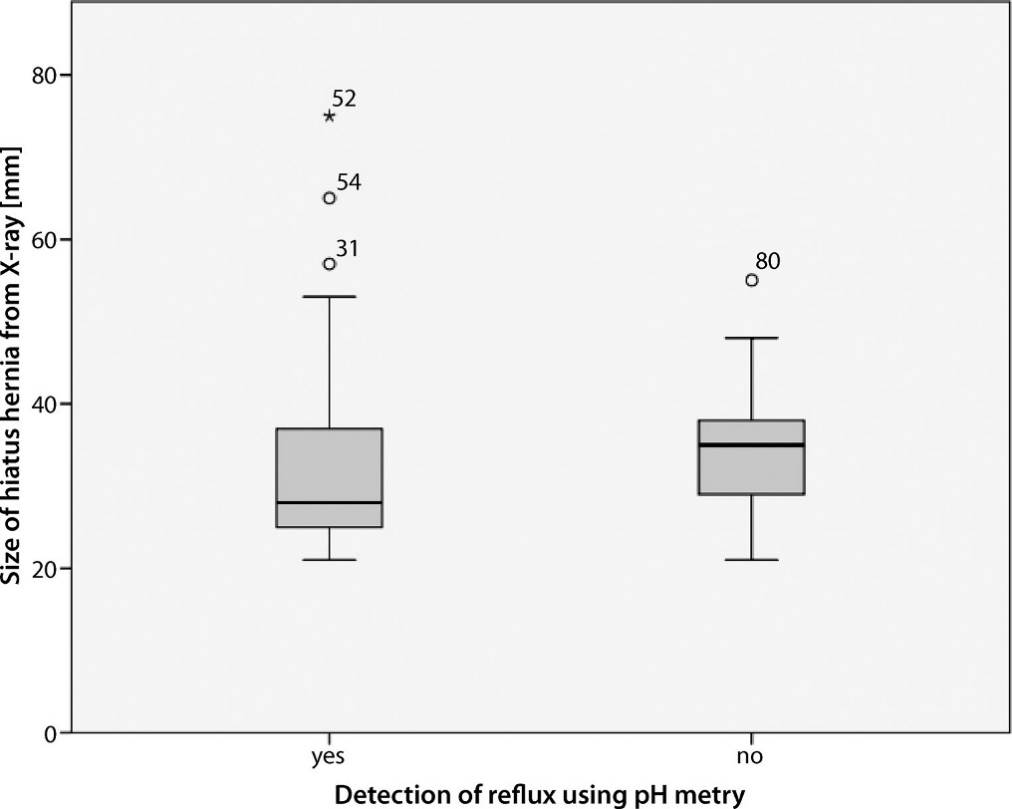
Box-plot diagram of radiographic hiatus defect size and pathological ambulatory multichannel intraluminal impedance-pH monitoring

The HH detection rate with barium swallow X‑ray also showed no correlation to objective GERD (Spearman: *p* = 0.370). There was also no significant correlation between radiographic hernia sizes and pathological MII (*p* = 0.177) as compared with HRM and MII. Interestingly, the radiologically measured mean HH size in patients with pathological acid reflux in MII evaluation was lower than the mean HH size in patients without pathological MII evaluation (32.13 mm vs. 35.04 mm) (Fig. 5).

An association between an abnormal GEFV/HH and pathological MII measurements could be demonstrated (Spearman: *p* = 0.047). Analysis of demographic data showed that obesity (BMI ≥30 kg/m^2^) was more frequently associated with GERD. All comparisons and demographics, as well as the accordance of HH detection rates and their correlation to GERD, are summarized in Table 1 and 2, respectively.

**Table 1:** Accordance of hiatal hernia rates by high resolution manometry, barium swallow X‑ray, and endoscopy

*n* = 112	HRM	Barium swallow X‑ray	Endoscopy
*HH Detection rate*			
HRM–X-ray	35/112 (31.25%) *No correlation*	86/112 (76.78%) *No correlation*	–
HRM–endoscopy	35/112 (31.25%) ***p < 0.001***	–	54/112 (48.20%) ***p < 0.001***
X-ray–endoscopy	–	86/112 (76.78%) *No correlation*	54/112 (48.20%) *No correlation*

*HRM *High-resolution manometry, *HH* hiatal hernia.

Statistical methods used: Kappa test for accordance; McNemar test for discordance of paired samples; Spearman’s rank correlation test

**Table 2:** Correlations of hiatal hernia (HH) rates and demographics to objective gastroesophageal reflux disease (GERD)

*n* = 112	HRM	Barium swallow X‑ray	Endoscopy
*HH size*			
HRM–GERD	GERD: 32.4 mm vs. NO GERD: 25.8 mm ***p*** **= 0.031**	–	–
X-ray–GERD	–	GERD: 32.13 mm vs. NO GERD: 35.04 mm *No correlation*	–
*HH/GEFV*			
HRM–GERD	28/35 (80%) *No correlation*	–	–
X-ray–GERD	–	63/86 (73.25%) *No correlation*	–
Endoscopy–GERD	–	–	46/54 (85.18%) ***p = 0.047***
*Demographics*			
Female gender (*n* = 52)	*No correlation*		
Age >60 (*n* = 41)	*No correlation*		
Body mass index 30 kg/m^2^ (*n* = 29)	***p = 0.023***		

*HRM *High-resolution manometry, *GERD* gastroesophageal reflux disease, *GEFV* gastroesophageal flap valve

Statistical methods used: Kappa test for accordance; McNemar for discordance of paired samples; Spearmans rank correlation test; non parametric Mann-Whitney-U test

## Discussion

Although the gold standard for diagnosing HH remains unclear, barium swallow X‑ray is considered to be the most sensitive tool [1, 5, 13, 14]. It was unexpected that the detection rates of HH and abnormal GEFV varied largely between the available diagnostic methods. Notably, the HH detection rate diversified widely between barium swallow X‑ray and the other two methods compared. The HH detection rate in barium swallow X‑ray examination was by far the highest. This extremely high detection rate of HH in radiographic examinations is remarkable, since manometric HH and endoscopically abnormal GEFV/HH findings were more or less half as much. This is an interesting fact, since some authors advocate that endoscopy is more sensitive than barium swallow X‑ray, and radiography can be omitted as a basic diagnostic test before intervention [19]. A limitation of this study is that it is not possible to say whether or how many false-positive findings are included in the barium swallow X‑ray examination, since no intraoperative findings are available for comparison. However, it is imaginable that radiographic evaluation may overestimate hiatal defect sizes. Previous studies showed that a surgeon cannot rely on the preoperative findings of the barium swallow X‑ray examination, since no correlation to intraoperative defect size measurements could be found [20]. The barium swallow X‑ray as a common procedure and even “gold standard” for preoperative HH assessment must be challenged according to the study results presented here, since there was no accordance either with objective GERD in MII or with manometric hiatal defect size measurements and evidence of endoscopic abnormal GEFV/HH. Despite considerable variation in the detection rate of HH, there was significant accordance between endoscopic and HRM findings. The status and accuracy of HRM interpretation of HH is as yet undetermined [15, 16]. The results presented here suggest that HRM hernia findings showed significant correlations with endoscopic evidence of abnormal GEFV/HH. Furthermore, they underline the findings of Khajanchee et al. that a negative result for HH by either HRM or endoscopy mandates additional testing [16].

Reliable preoperative assessment of HH is crucial in proper patient selection for different treatment options. Patients with unsuccessful conservative treatment of GERD and without evidence of HH may be candidates for endoscopic treatments such as full-thickness gastroplication [21] or radiofrequency energy delivery (Stretta procedure), which has been shown in several studies to improve GERD symptoms and quality of life for approximately two thirds of patients [22]. Other treatment options for patients with diagnosed HH include augmentation of the lower esophageal sphincter barrier with a magnetic device using a standard laparoscopic approach [23], or laparoscopic fundoplication according to Nissen or Toupet with/without mesh augmentation depending on the size of the hiatal defect [24].

Furthermore, the results of this study underline the importance of the GEFV in the pathophysiology of GERD. As in previous studies [25–27], this study found a significant accordance between endoscopic grading and GERD. While it is relatively typical for HH to have a Hill 3 or 4 valve, it is also possible but unusual to have a Hill 3 or 4 valve with no HH. Therefore, not only Hill grading but also axial displacement (separation of squamocolumnar junction = Z‑line from diaphragmatic impression >2 cm) were routinely evaluated to assess real HH.

HRM [15, 16] may offer advantages over conventional methods, including improved identification of motility disorders, outflow obstruction, and even HH, and ease interpretation. This technique is capable of displaying spatial and topographic pressure profiles of the gastroesophageal junction and crural diaphragm in real time. It is not clear whether manometry studies should better be done supine, upright, or both. It is not possible to rule out the possibility that one or the other might affect the number of “sliding” hernias detected. The investigations discussed here were performed in a supine position with 30° upper body elevation. Nevertheless, the results of this study seem to underline the role of HRM examinations in patients with GERD symptoms, since it was possible to demonstrate a significant correlation between manometrically measured HH size and objective GERD.

Both endoscopy and HRM represent in vivo dynamic evaluation tools of the esophagogastric junction, and may thus demonstrate an improved correlation profile with objective GERD, although the results of a recent study suggest that the two tests had high discordance and both showed high false-negative results in the detection of HH [16]. On the other hand, barium swallow X‑ray studies may provide a fragmentary anatomical view of the functional activity of the esophagogastric junction. The variety between HRM, endoscopy, and radiographic barium swallow regarding the detection rates of HH and abnormal GEFV, respectively, warrant further examination and comparison with the intraoperatively objective determination of HH.

In conclusion*, *a reliable assessment of HH is essential for proper patient selection prior to GERD treatment. Despite considerable variation in the detection rate of HH, there was significant accordance between endoscopic and HRM findings. Interestingly, only endoscopically assessed GEFV/HH but no HH detection rates in HRM and X‑ray showed a correlation profile to objective GERD. Furthermore, barium swallow X‑ray showed neither a correlation to HRM in terms of HH detection nor to abnormal GEFV. However, barium swallow X‑ray provided the highest rate of HH detection and, for the reliable exclusion of HH prior to treatment, all three investigations mentioned seem to be necessary in order of low conformity.

## Abbreviations

HH: Hiatus Hernia
GERD: Gastroesophageal reflux disease
GEFV: Gastroesophageal flap valve
HRM: High-resolution manometry
LES: Lower esophageal sphincter
MII: Ambulatory multichannel intraluminal impedance-pH monitoring
PPI: Proton pump inhibitors
